# TMEM45A is essential for hypoxia-induced chemoresistance in breast and liver cancer cells

**DOI:** 10.1186/1471-2407-12-391

**Published:** 2012-09-06

**Authors:** Lionel Flamant, Edith Roegiers, Michael Pierre, Aurélie Hayez, Christiane Sterpin, Olivier De Backer, Thierry Arnould, Yves Poumay, Carine Michiels

**Affiliations:** 1 URBC-NARILIS, University of Namur-FUNDP, 61 rue de Bruxelles, 5000, Namur, Belgium; 2 Molecular Biology Research Unit (URBM)-NARILIS, University of Namur-FUNDP, 61 rue de Bruxelles, 5000, Namur, Belgium; 3 URPhyM-NARILIS, University of Namur-FUNDP, 61 rue de Bruxelles, 5000, Namur, Belgium

**Keywords:** Chemoresistance, Cancer, Gene expression, Hypoxia, Apoptosis, Microarrays

## Abstract

**Background:**

Hypoxia is a common characteristic of solid tumors associated with reduced response to radio- and chemotherapy, therefore increasing the probability of tumor recurrence. The aim of this study was to identify new mechanisms responsible for hypoxia-induced resistance in breast cancer cells.

**Methods:**

MDA-MB-231 and HepG2 cells were incubated in the presence of taxol or etoposide respectively under normoxia and hypoxia and apoptosis was analysed. A whole transcriptome analysis was performed in order to identify genes whose expression profile was correlated with apoptosis. The effect of gene invalidation using siRNA was studied on drug-induced apoptosis.

**Results:**

MDA-MB-231 cells incubated in the presence of taxol were protected from apoptosis and cell death by hypoxia. We demonstrated that TMEM45A expression was associated with taxol resistance. TMEM45A expression was increased both in MDA-MB-231 human breast cancer cells and in HepG2 human hepatoma cells in conditions where protection of cells against apoptosis induced by chemotherapeutic agents was observed, i.e. under hypoxia in the presence of taxol or etoposide. Moreover, this resistance was suppressed by siRNA-mediated silencing of TMEM45A. Kaplan Meier curve showed an association between high TMEM45A expression and poor prognostic in breast cancer patients. Finally, TMEM45 is highly expressed in normal differentiated keratinocytes both in vitro and in vivo, suggesting that this protein is involved in epithelial functions.

**Conclusion:**

Altogether, our results unravel a new mechanism for taxol and etoposide resistance mediated by TMEM45A. High levels of TMEM45A expression in tumors may be indicative of potential resistance to cancer therapy, making TMEM45A an interesting biomarker for resistance.

## Background

The development of therapy resistance continues to be a major problem in the treatment of patients with cancer. Treatment failure has been very recently identified as one of the four major issues in cancer research [1]. Identification of underlying mechanisms is thus of great value.

Some mechanisms underlying cancer resistance to chemotherapy have been unraveled [2]. One of the well characterized cellular factors of resistance is the overexpression of the P-glycoprotein encoded by the MDR1 gene [3]. This protein is an efflux pump that expulses the chemotherapeutic drug out of the tumor cells. Other efflux pumps have been identified, all belonging to the ABC (ATP-binding cassette) transporter family, which expression may also play a role in inducing chemoresistance [4]. Numerous molecules inhibiting efflux pump activity have been tested but without real therapeutic success or with unacceptable toxicity [5]. Other important causes of resistance are the molecular alterations of the drug targets. Other resistance mechanisms include enhanced DNA repair, loss of p53, inhibition of apoptosis, activation of cell survival pathways caused by mutations or epigenetic alterations occurring in the context of genetic instability (selection of resistant cells) [6]. There are however still numerous open questions regarding the mechanisms allowing cancer cells to escape drug-induced toxic effects.

Tumor hypoxia is often associated with resistance to chemotherapy [7] and radiotherapy [8], with tumor progression, aggressiveness and metastasis, and therefore with an increased probability of tumor recurrence [9]. Identification of the mechanisms responsible for this protection would therefore have significant clinical benefits.

Hypoxia, the reduction of the normal level of tissue oxygen tension, is a common feature of solid tumors caused by of the abnormal vascular network and the high proliferation rate of cancer cells [10]. Intratumoral hypoxia develops when cells are located further than 100–180 μm from a functional blood vessel. Indeed, oxygen is unable to diffuse beyond this distance from a capillary before it is completely metabolized.

It was shown that up to 50-60% of locally advanced solid tumors may exhibit hypoxic and/or anoxic tissue areas heterogeneously distributed within the tumor mass [11]. Hypoxia occurs in breast tumors, as in other solid tumors, mostly because of tumor outgrowing of the existing vasculature (reviewed in [12]). In breast cancer, hypoxia has been correlated with bad prognosis. Indeed, HIF-1α [13] or HIF-2α [14] expression, as surrogate markers of tumor hypoxia, correlates with distant recurrence and poor outcome. Furthermore, expression profiles of the hypoxic markers, GLUT1 and CAIX, also correlate with adverse prognostic factors in breast cancer [15]. Hepatocellular carcinomas were also reported to display hypoxia [16]. Hypoxic regions have also been identified in tumors of many other histotypes such as brain tumors [17,18], head and neck [19] or cervical [20], endometrial [21] and lung [22] cancers.

It is now thought that hypoxia, according to its severity, can either promote apoptosis and cell death or contrariwise induce cell growth and survival by provoking an adaptive response. To survive in the hypoxic environment which takes place in the centre of solid tumors, cells co-opt adaptive mechanisms leading to a variety of biological responses, i.e. switching to a glycolytic metabolism, proliferation, evasion of apoptosis, obtaining a limitless replicative potential, induction of angiogenesis, invasion of the immune system and tissue invasion and metastasis [23].

If some mechanisms underlying the hypoxia-induced radio- and chemoresistance begin to be understood, the actual actors of the protection still need to be identified. The aim of this study was to characterize the mechanisms underlying the hypoxia protection against paclitaxel-induced apoptosis observed in MDA-MB-231 cells, but also the hypoxia protection against etoposide-induced apoptosis previously observed in HepG2 cells [24,25].

We exposed MDA-MB-231 cells to two chemotherapeutics agents, paclitaxel and epirubicin, used as apoptosis inducers. These two drugs belong respectively to the class of taxoids and anthracyclines, which are considered to be the most active agents in the treatment of breast cancer [26]. Paclitaxel binds to microtubules and causes their stabilization, inducing cell cycle arrest at G2/M mitotic phase followed by apoptosis. It also induces modulation of the expression or posttranslational modification of pro- and anti-apoptotic proteins [27]. Epirubicin HCl, a derivative of doxorubicin, leads to the inhibition of DNA and RNA synthesis by intercalation between base pairs of the DNA/RNA. It also inhibits topoisomerase II by stabilizing DNA-topoisomerase complex, resulting in DNA damage and induction of apoptosis and cell death [28]. It was also proposed that epirubicin could induce formation of reactive oxygen species promoting apoptosis [29]. As a second experimental model, we used etoposide to induce cell death in HepG2 cells. This antineoplastic drug functions as a topoisomerase II inhibitor, hence inducing double strand breaks in DNA, leading to the activation of apoptosis in a p53 dependent manner [30]. The biochemical and molecular mechanisms of apoptosis activation by these drugs are complex and are still under investigation.

We recently highlighted two mechanisms by which hypoxia protects cells from apoptosis via the activation of hypoxia-inducible factor-1 (HIF-1) and AP-1 transcription factors and the consecutive changes in expression of anti- and pro-apoptotic proteins [31]. Here, we investigated other changes in gene expression that may explain how hypoxia exerts its protective effect against paclitaxel induced-apoptosis.

## Results

### Hypoxia protects MDA-MB-231 cells against paclitaxel-induced apoptosis

**Figure 1:**
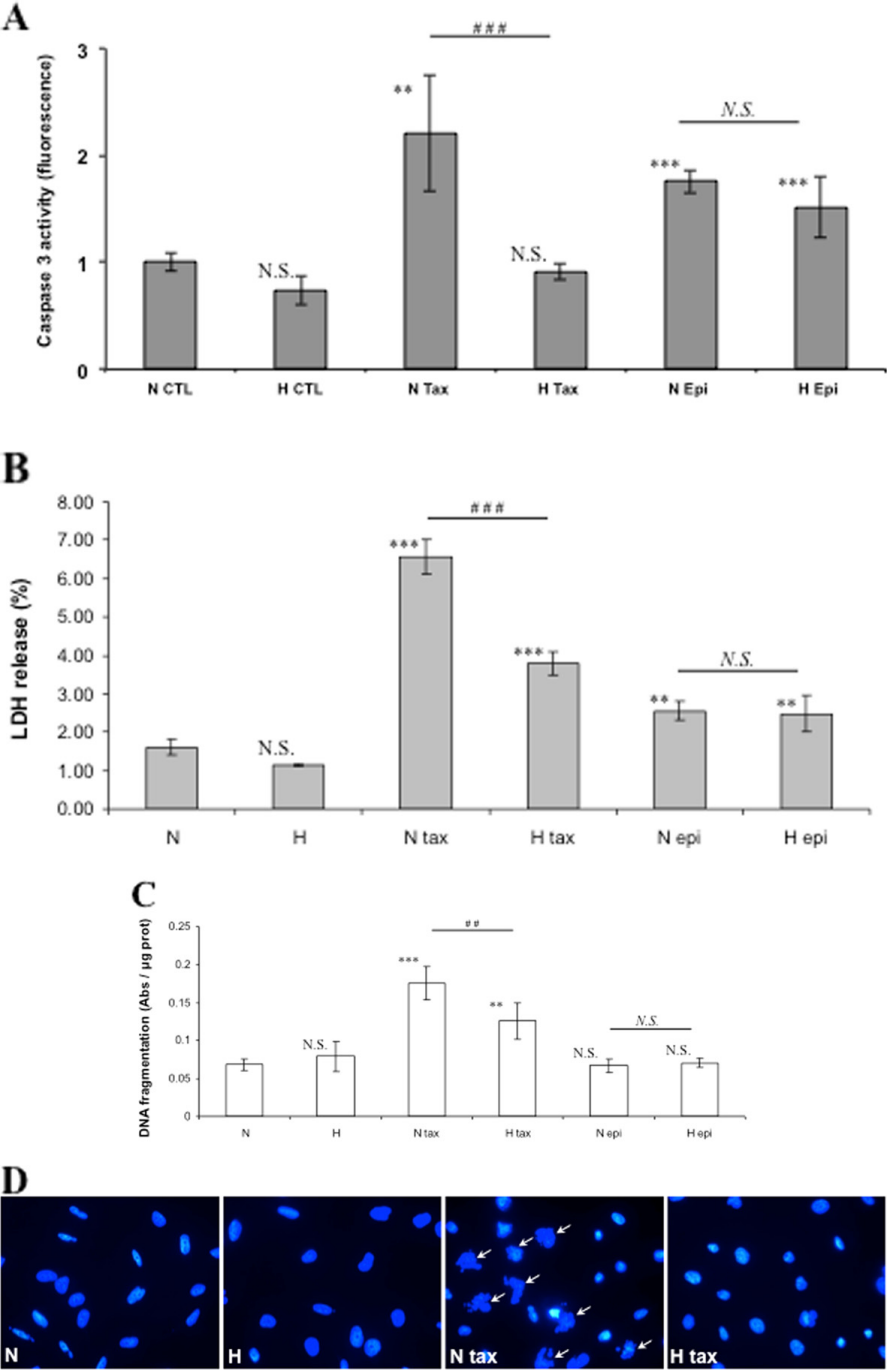
**Effect of hypoxia on paclitaxel or epirubicin-induced apoptosis and cell death.** MDA-MB-231 cells were incubated under normoxic (N) or hypoxic (H) conditions with or without paclitaxel (tax, 50 μM) or epirubicin (epi, 10 μM) for 16 hours. (**A**) Caspase 3 activity was assayed by measuring free AFC released from the cleavage of the caspase 3 specific substrate Ac-DEVD-AFC. Results are expressed in fluorescence intensity, as mean ± 1 SD (n = 3). (**B**) After the incubation, LDH release was assessed. Results are expressed as mean ± 1 SD (n = 3). (**C**) After the incubation, DNA fragmentation was assayed using an ELISA for soluble nucleosomes (Cell Death Detection Elisa, Roche). Results are expressed as mean ± 1 SD (n = 3). (**D**) Nuclear fragmentation was observed after nuclei labeling with DAPI using non-confocal fluorescent microscope (40x magnification). Fragmented nuclei are pointed by arrows. N.S. = non significantly different from control, ** = significantly different from control (p < 0.01), *** = significantly different from control (p < 0.001) ; *N.S.* = no significant difference between N epi and H epi, ## = significant difference between N tax and H tax (p < 0.01). ### = significant difference between N tax and H tax (p < 0.001).

Recent studies showed that hypoxia confers resistance against chemotherapy-induced apoptosis in cancer cells [32,33]. We have shown previously that hypoxia protects human breast cancer MDA-MB-231 cells against paclitaxel-induced apoptosis [31]. To confirm the effect of hypoxia on paclitaxel- and epirubicin-induced apoptosis, we assessed caspase 3 activity and cell death in MDA-MB-231 cells incubated with these drugs under normoxia or hypoxia (Figure 1A, B). 50 μM paclitaxel and 10 μM epirubicin were chosen, from concentration titration curve, to induce a marked induction of apoptosis in order to be able to observe a protective effect but not a too high induction of ell death that could no longer be inhibited (data not shown). Hypoxia per se did not induce apoptosis or cell death since no increase in caspase 3 activity or LDH release was observed. Paclitaxel and epirubicin triggered apoptosis and cell death as shown by an increase in caspase 3 activity and in LDH release in normoxia. Hypoxia did not modify epirubicin-induced apoptosis or cell death. However, it markedly inhibited paclitaxel-induced increase in caspase 3 activity and LDH release. Hypoxia also inhibited paclitaxel-induced increase in caspase 3 activity at 100 μM (data not shown).

DNA fragmentation was also assessed. An increase in DNA fragmentation was observed in the presence of paclitaxel under normoxia, which was significantly decreased by hypoxia (Figure 1C). No effect of epirubicin was detected, probably because an incubation of 16 hours was not long enough to induce the later stages of apoptosis. Nucleus morphology was observed in fluorescence microscopy after cell labelling with DAPI. Fragmented nuclei were observed in cells exposed to paclitaxel under normoxia but neither in cells exposed to paclitaxel under hypoxia, nor in cells that were not incubated with paclitaxel (Figure 1D).

Altogether, these data demonstrated that hypoxia was able to protect MDA-MB-231 cells against the paclitaxel-induced apoptosis.

### Hypoxia combined with paclitaxel or epirubicin induces changes in gene expression

In order to identify the mechanism(s) responsible for the hypoxia-induced protection, Affymetrix whole transcriptome analysis was performed using RNA isolated from control cells and from cells incubated in the presence of taxol or epirubicin, both under normoxia and hypoxia. Experimental triplicates were analyzed for each experimental condition.

Unsupervised hierarchical clustering was performed without any gene selection in order to group the conditions on the basis of their similarity measured over all probe sets of the array (Additional file 1: Figure S1). Unsupervised cluster analysis using centered correlation and average linkage showed that control cells were well separated from drug-treated cells. Moreover, for control cells and cells incubated in the presence of taxol, hypoxia was separated from normoxia while it was not the case for epirubicin-treated cells. These results are in good accordance with the protection of drug-induced apoptosis brought by hypoxia for paclitaxel but not for epirubicin.

We hypothesized that gene expression modifications in the different conditions could influence the paclitaxel-induced apoptosis, leading to cell protection in hypoxia. We thus compared differences and similarities in gene expression modifications in these conditions and genes were sorted to select only those with a high difference in expression between cells exposed to paclitaxel in normoxia (“N tax”) and cells exposed to paclitaxel under hypoxia (“H tax”), which could therefore be involved in the hypoxia-induced protection against paclitaxel-induced apoptosis.

First of all, the probe sets were ranked in ascending order of the p values of their differential expression between “N tax” and “H tax”, and only highly significant ones with a p value lower than 0.005 were selected. A second round of selection of probe sets was performed by keeping only those with a minimum fold change of at least 4 times between these 2 conditions. Table 1 shows the 31 probe sets selected on the basis of their p value and fold change between “N tax” and “H tax”.

**Table 1:** Gene expression profiling in MDA-MB-231 cells incubated with or without paclitaxel (tax) or epirubicin (epi) under normoxic (N) or hypoxic (H) conditions

	**Fold Induction**	**Standard Deviation**	**Fold Induction**	**Standard Deviation**	**Fold Induction**	**Standard Deviation**	**Fold Induction**	**Standard Deviation**	**Fold Induction**	**Standard Deviation**	**Fold Induction**	**Standard Deviation**	**H tax / N tax**	***P values***	**H epi / N epi**
	**N**		**H**		**N tax**		**H tax**		**N epi**		**H epi**			***H tax vs N tax***	
SPAG4	1	0.23	84.19	0.07	0.73	0.14	38.57	0.17	1.86	0.07	1.47	0.43	52.76	*4.88E-05*	0.79
ALDOC	1	0.39	91.10	0.17	0.46	0.07	22.20	0.23	0.73	0.16	0.86	0.25	48.31	*0.0002552*	1.18
ANGPTL4	1	0.03	53.47	0.19	0.80	0.19	26.54	0.35	0.67	0.04	0.69	0.00	33.10	*0.0006633*	1.02
PFKFB4	1	0.12	12.11	0.05	0.51	0.03	11.73	0.15	***0.04***	***0.09***	***0.04***	***0.01***	23.12	*0.0001621*	0.94
C10orf10 **probe set 1**	***1***	***0.15***	***69.43***	***0.30***	***0.86***	***0.03***	***18.27***	***0.43***	***0.87***	***0.03***	***0.87***	***0.01***	***21.19***	*0.0019277*	1.00
TMEM45A	1	0.59	106.19	0.01	5.13	0.29	86.65	0.21	8.80	0.31	10.01	0.18	16.88	*0.0002627*	1.14
RASSF4 **probe set 1**	1	0.09	11.21	0.15	0.40	0.02	5.86	0.32	0.15	0.74	0.06	0.29	14.65	*0.0004547*	0.42
ANKRD37	1	0.18	21.08	0.15	1.92	0.14	21.56	0.23	0.19	0.70	0.14	0.10	11.26	*0.0001464*	0.71
PDK1 **probe set 1**	1	0.05	8.58	0.13	1.12	0.03	9.01	0.26	3.03	0.07	3.34	0.04	8.06	*0.0004021*	1.10
PDK1 **probe set 2**	1	0.18	9.08	0.27	1.36	0.16	10.98	0.40	4.13	0.24	3.56	0.04	8.05	*0.0039759*	0.86
BNIP3 **probe set 1**	1	0.05	10.33	0.13	1.39	0.07	10.62	0.20	1.85	0.09	1.87	0.13	7.66	*0.0004483*	1.01
NDRG1	1	0.21	10.01	0.08	1.23	0.15	9.28	0.12	0.35	0.23	0.27	0.07	7.51	*6.10E-05*	0.78
RASSF4 **probe set 2**	1	0.03	9.04	0.10	0.73	0.02	5.05	0.37	0.79	0.06	0.81	0.15	6.91	*0.0023704*	1.03
AK3L1	1	0.04	3.91	0.07	0.36	0.08	2.26	0.08	1.84	0.05	2.34	0.06	6.32	*0.0003084*	1.27
WFDC3	1	0.23	8.94	0.31	0.39	0.24	2.46	0.12	0.53	0.37	1.04	0.33	6.28	*0.0001468*	1.95
P4HA1	1	0.14	10.23	0.05	1.00	0.09	5.60	0.17	1.66	0.15	1.56	0.15	5.59	*0.0001797*	0.94
MALL	1	0.11	2.64	0.16	0.22	0.19	1.23	0.09	1.07	0.24	1.15	0.12	5.54	*0.0001154*	1.07
RAB20	1	0.09	4.65	0.02	0.63	0.19	3.33	0.22	0.16	0.23	***0.08***	***0.03***	5.32	*0.0003267*	0.48
EGLN3	***1***	***0.02***	***13.74***	***0.17***	***1.01***	***0.00***	***5.14***	***0.35***	***0.94***	***0.09***	***1.00***	***0.02***	***5.09***	*0.0034448*	1.06
FUT11	1	0.23	5.60	0.12	0.49	0.22	2.46	0.16	0.32	0.30	0.21	0.35	5.02	*0.0037602*	0.67
BNIP3 **probe set 2**	1	0.09	6.22	0.07	1.25	0.09	6.18	0.13	1.48	0.08	1.58	0.08	4.93	*4.93E-05*	1.07
PLOD2	1	0.04	7.09	0.11	1.13	0.05	5.45	0.25	2.83	0.20	3.10	0.03	4.83	*0.0005200*	1.10
PGF	1	0.18	12.21	0.05	0.61	0.22	2.87	0.11	0.39	0.30	0.22	0.25	4.69	*0.0008395*	0.56
ENO2	1	0.11	5.46	0.06	0.90	0.13	4.17	0.08	2.01	0.10	2.32	0.09	4.66	*0.0001298*	1.15
TNFSF10	1	0.13	4.11	0.19	0.90	0.52	4.15	0.36	0.98	0.14	1.01	0.09	4.63	*0.0026329*	1.03
ZNF395	1	0.20	3.30	0.03	0.41	0.25	1.84	0.21	0.78	0.20	0.83	0.20	4.49	*0.0009758*	1.06
PFKFB3	1	0.00	5.50	0.10	1.17	0.11	5.24	0.32	0.59	0.27	0.33	0.14	4.47	*0.0019571*	0.56
ANKZF1	1	0.11	6.70	0.07	1.31	0.16	5.77	0.17	0.59	0.15	0.58	0.10	4.40	*0.0012567*	0.98
JMJD1A	1	0.11	5.36	0.10	2.12	0.02	8.93	0.19	0.35	0.98	0.15	0.22	4.21	*0.0017354*	0.42
C10orf10 **probe set 2**	***1***	***0.01***	***23.38***	***0.78***	***1.00***	***0.00***	***4.12***	***0.34***	***1.01***	***0.01***	***1.37***	***0.45***	***4.14***	*0.0020200*	1.36
IL24	1	1.42	0.14	0.20	5.70	0.27	0.82	0.60	6.86	1.43	1.07	0.25	0.14	*0.0010651*	0.16

Cells were incubated in the different conditions for 16 hours before RNA extraction, target preparation, hybridization to Affymetrix HG-U133 Plus 2.0 arrays, washing and array signal acquisition, as described in Material an Methods. Each value of the fold induction columns is the mean of three ratio values calculated from three independent experiments. These 31 candidate genes were selected on the basis of their p value (p < 0.005) and fold change (at least 4 times) between “N tax” and “H tax”. They are italicized if qualitative data (as discussed in Material and Methods).

Some of the selected genes were already known to be upregulated by hypoxia such as ALDOC, PFKFB4, PDK1, BNIP3, NDRG1, ENO2, PFKFB3 and JMJD1A. The expression patterns of 5 genes were validated by qRT-PCR: for 4 of them (TMEM45A, STC1, FAM26F and GDF15), the changes in mRNA levels were confirmed. However, no change was detected for S100A10 by real-time RT-PCR (data not shown).

**Figure 2:**
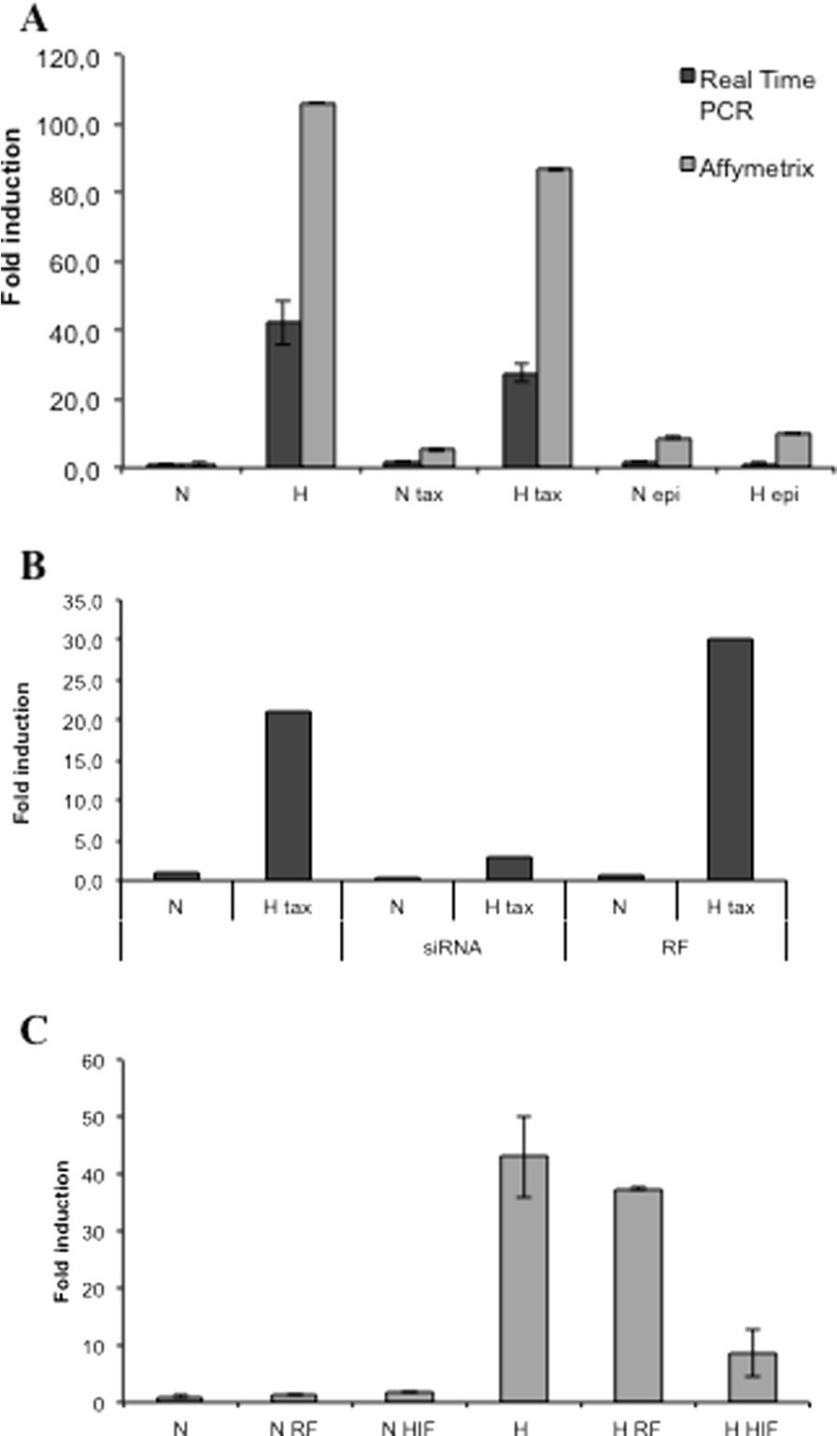
**Analysis of TMEM45A gene expression level obtained with real time PCR and Affymetrix microarrays and effect of TMEM45A silencing on TMEM45A expression.** (**A**) For Affymetrix microarray results: cells were incubated under normoxic (N) or hypoxic (H) conditions with or without paclitaxel (tax, 50 μM) or epirubicin (epi, 10 μM) for 16 hours before total RNA extraction. Results are expressed as mean ratios indicating a fold-increase or decrease in gene expression by comparison with the reference condition (normoxia) ± 1 SD (n = 3). For Real Time PCR results: after incubation, total RNA has been extracted and retro-transcribed in cDNA. A real time PCR has been performed with specific primers for TMEM45A and for RPL13A, a house-keeping gene. Results are expressed as mean of induction level by comparison with the reference condition (normoxia) ± 1 SD (n = 3). (**B**) Effect of TMEM45A silencing on TMEM45A expression. MDA-MB-231 cells were transfected 24 h with TMEM45A siRNA (siRNA) or RISC-free control siRNA (RF) (50 nM). Cells were then incubated in normoxia or hypoxia with paclitaxel (tax, 50 μM) for 16 hours. TMEM45A mRNA expression was quantified by real-time RT-PCR using RPL13A as the house-keeping gene. Results are expressed in induction level by comparison with the reference condition, normoxia. (**C**) Effect of HIF-1α silencing on TMEM45A expression. MDA-MB-231 cells were transfected 24 h with HIF1A siRNA (HIF) or RISC-free control siRNA (RF) (50 nM). Cells were then incubated in normoxia or hypoxia for 16 hours. TMEM45A mRNA expression was quantified by real-time RT-PCR using RPL13A as the house-keeping gene. Results are expressed in induction level by comparison with the reference condition, normoxia, as means ± 1 SD (n = 3).

Interestingly, level of TMEM45A mRNA was increased more than 16 fold in cells exposed to paclitaxel under hypoxia compared to cells exposed to paclitaxel under normoxia and was increased in hypoxic condition with or without paclitaxel but not in the presence of epirubicin (Figure 2A). This expression profile was parallel to the apoptotic profile of cells exposed to these two drugs under normoxia or hypoxia. This suggested that this protein could play a role in modulating drug-induced apoptosis. TMEM45A encodes a hypothetical transmembrane protein with unknown function. We thus decided to investigate further this gene.

### TMEM45A is essential to mediate the hypoxia-induced protection against paclitaxel-induced apoptosis

In order to test the possible role of TMEM45A in the hypoxia-induced resistance to taxol, its expression was knocked down in MDA-MB-231 using siRNA. A concentration of 50 nM of siRNA was high enough to strongly inhibit the mRNA expression of TMEM45A while the RISC-Free negative control siRNA had no effect (Figure 2B). TMEM45A has been described to be a HIF-1 target gene in different types of cancer cells [34]. We verified that it was also the case in MDA-MB-231 cells. HIF-1α silencing by siRNA confirmed the role of HIF-1α since a decreased by more than 80% in the hypoxia-induced upregulation of TMEM45A mRNA expression was observed (Figure 2C).

It has to be noted that numerous experiments aimed to analyze TMEM45A protein expression by western blot have been performed. Whatever the lysis buffer and the conditions of antibody incubation with the membrane, we did not succeed to detect a band corresponding to the protein (i.e. that is no longer observed in extracts from cells transfected with the siRNA targeting TMEM45A mRNA). However, we validated the Sigma anti-TMEM45A antibody using two approaches: (i) TMEM45A has been immunoprecipitated from post-confluent keratinocyte protein extract (see below), using the anti-TMEM45 antibody. Proteins that were immunoprecipitated were separated by SDS-PAGE and the gel area containing proteins between 64 and 98 kDa (i.e. the approximate apparent molecular weight range observed for the protein in western blot analysis of extract from the same cell type) was excised after silver staining of proteins. Proteins were digested with trypsin, extracted from the polyacrylamide gel and analyzed by mass spectrometry. TMEM45A was identified in this band, while it was not present in bands corresponding to negative controls. (ii) TMEM45A cDNA was cloned into the pDest475 plasmid allowing the expression of a HA-tagged version of TMEM45A. This plasmid was transfected into MCF-7 cells and cells have been analyzed by immunofluorescence staining using an anti-HA and the anti-TMEM45A antibody. Results showed that both antibodies recognized the same vesicular structures within transfected cells and clearly co-localized (data not shown). No labeling was observed in untransfected cells with either of the antibodies (data not shown). From these results, we concluded that the anti-TMEM45A antibody from Sigma does recognize specifically the TMEM45A protein. It is able to detect the protein when expressed in high quantity (upon artificial overexpression or in post-confluent keratinocytes (ct in real-time PCR = 21–26 with 21 for post-confluent and 26 for subconfluent keratinocytes) but not in cells with mild expression (MDA-MB-231 (ct in real-time PCR = 31) or HepG2 cells ((ct in real-time PCR = 29)), possibly because it has a low affinity.

**Figure 3:**
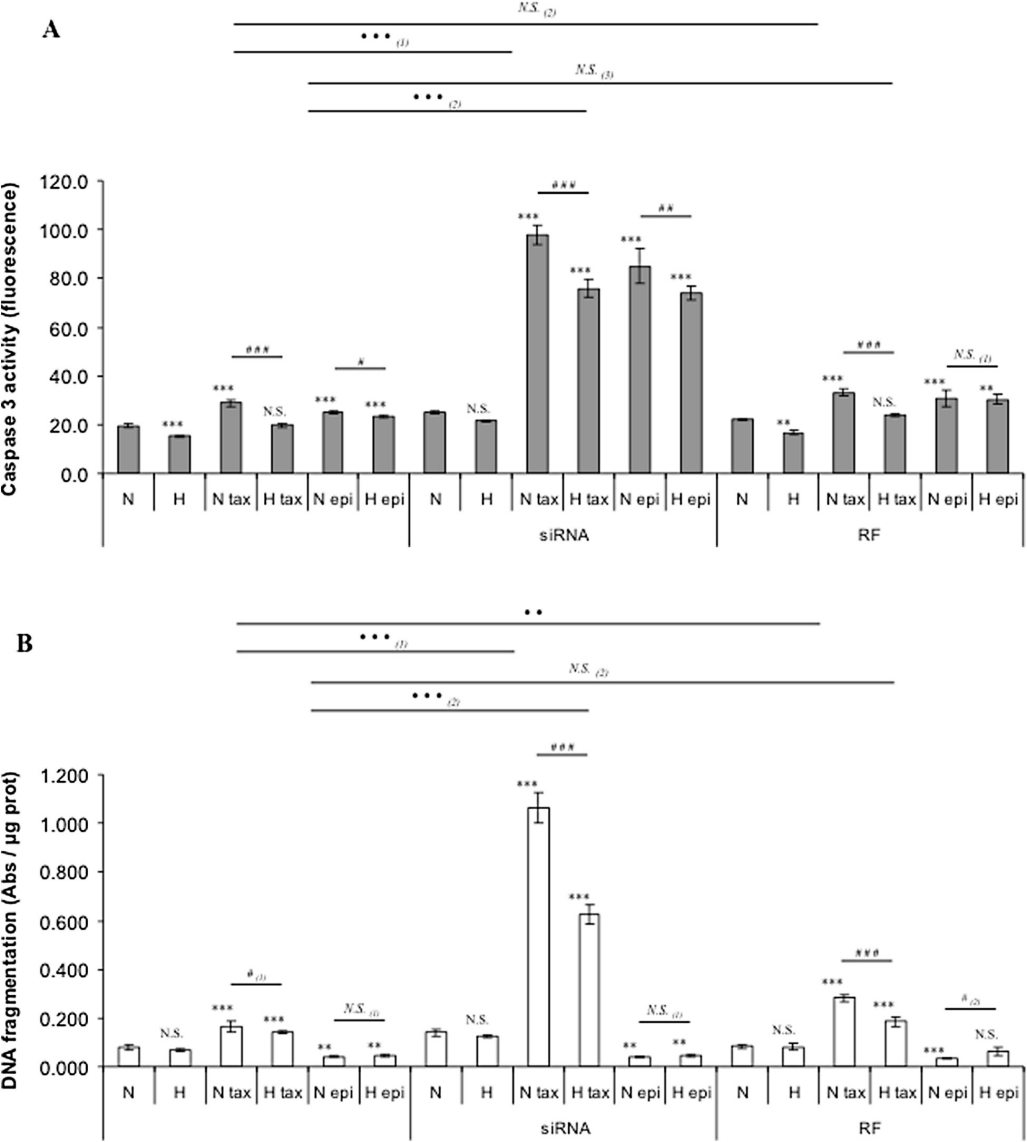
**Effect of TMEM45A silencing on the protective effect of hypoxia on paclitaxel-induced apoptosis.** 8 h post transfection with TMEM45A siRNA (siRNA) or RISC-free control siRNA (RF) (50 nM, 24 h), MDA-MB-231 cells were incubated under normoxic (N) or hypoxic (H) conditions with or without paclitaxel (tax, 50 μM) or epirubicin (epi, 10 μM) for 16 hours. (**A**) After transfection and incubation, caspase 3 activity was assayed by measuring free AFC released from the cleavage of the caspase 3 specific substrate Ac-DEVD-AFC. Results are expressed in fluorescence intensity, as mean ± 1 SD (n = 3). (**B**) After the incubation, DNA fragmentation was assayed using an ELISA for soluble nucleosomes (Cell Death Detection Elisa, Roche). Results are expressed as mean ± 1 SD (n = 3). Statistical analyses were determined independently for the 3 subgroups *without siRNA*, *with anti-TMEM45A siRNA (siRNA)* and *with RISC-free control siRNA (RF)* ; N.S. = non significantly different from control (N, N siRNA or N RF), ** = significantly different from control (p < 0.01), *** = significantly different from control (p < 0.001) ; *N.S.*_(1)_ = no significant difference between N epi and H epi, #, ### = significant difference between _(1)_ N tax and H tax or _(2)_ N epi and H epi (p < 0.05 ; <0.001), *N.S*. _(2)_ = no significant difference between H tax and H tax RF, ·· = significant difference between N tax and N tax RF (p < 0.01), ··· = significant difference between _(1)_ N tax and N tax siRNA or _(2)_ H tax and H tax siRNA (p < 0.001).

We then investigated if TMEM45A was involved in the hypoxia-induced resistance to paclitaxel-induced apoptosis. For that purpose, cells were transfected with TMEM45A or control siRNA before being incubated under normoxia or hypoxia in the presence of one or the other chemotherapeutic agent. If TMEM45A plays an anti-apoptotic role, an increase in the paclitaxel-induced caspase 3 activity is expected when inhibited by siRNA. Figure 3A shows that caspase 3 activity was significantly increased in TMEM45A siRNA transfected cells incubated with paclitaxel or epirubicin, both under normoxia and hypoxia. The caspase 3 activity measured for TMEM45A siRNA transfected cells incubated with paclitaxel under hypoxia reached a level much higher than the activity measured for non transfected cells incubated with paclitaxel in normoxia, indicating additive effects of hypoxia and of TMEM45A for cell protection. No modification was observed in cells transfected with the negative control siRNA.

We confirmed these observations by determining the effect of TMEM45A silencing on paclitaxel induced DNA fragmentation. As observed in Figure 3B, the negative control siRNA did not influence DNA fragmentation, whereas TMEM45A siRNA transfection induced a strong and significant increase in DNA fragmentation in cells incubated with paclitaxel under normoxia or hypoxia compared to non transfected cells incubated in the same conditions.

These results clearly demonstrated for the first time an implication of TMEM45A as an anti-apoptotic protein.

### TMEM45A is also involved in the hypoxia-induced protection of HepG2 cells against etoposide-induced apoptosis

In order to further confirm the role of TMEM45A in drug resistance, we used another experimental model: HepG2 hepatocellular carcinoma cells exposed to etoposide. We have previously shown that hypoxia protected HepG2 human hepatoma cells against etoposide-induced apoptosis. HIF-1 and AP-1 were shown to participate to this protective effect [32]. Using siRNA targeting TMEM45A, we determined if TMEM45A could also be involved in the protection of HepG2 cells against apoptosis induced by etoposide under hypoxia.

**Figure 4:**
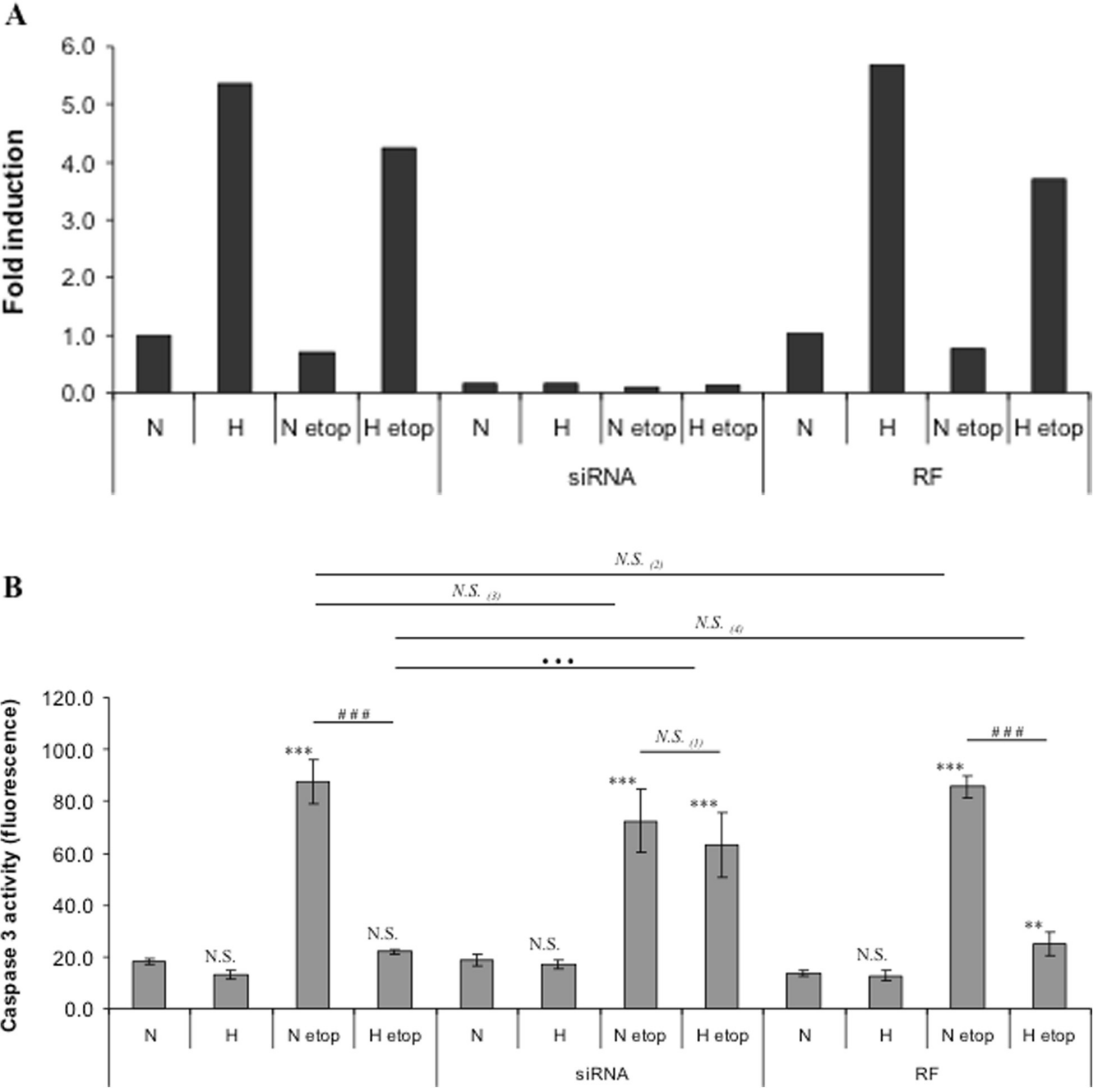
**Analysis of TMEM45A gene expression level and effect of TMEM45A silencing on TMEM45A expression and caspase 3 activity in HepG2 cells.** HepG2 cells were incubated under normoxic (N) or hypoxic (H) conditions with or without etoposide (etop, 50 μM) for 16 hours, 8 h post transfection with TMEM45A siRNA (siRNA) or RISC-free control siRNA (RF) (50 nM, 24 h). (**A**) After transfection and incubation, total RNA has been extracted and retro-transcribed in cDNA. A real time PCR has been performed with specific primers for TMEM45A and for RPL13A, a house-keeping gene. Results are expressed in induction level by comparison with the reference condition, normoxia. (**B**) After transfection and incubation, caspase 3 activity was assayed by measuring free AFC released from the cleavage of the caspase 3 specific substrate Ac-DEVD-AFC. Results are expressed in fluorescence intensity, as mean ± 1 SD (n = 3). Statistical analyses were determined independently for the 3 subgroups *without siRNA*, *with TMEM45A siRNA (siRNA)* and *with RISC-free control siRNA (RF)* ; N.S. = non significantly different from control (N, N siRNA or N RF), ** = significantly different from control (p < 0.01), *** = significantly different from control (p < 0.001) ; *N.S.*_(1)_ = no significant difference between N etop and H etop, ### = significant difference between N etop and H etop (p < 0.001), *N.S.* = no significant difference between _(2)_ N etop and N etop RF, _(3)_ N etop and N etop siRNA, or _(4)_ H etop and H etop RF, ··· = significant difference between H etop and H etop siRNA (p < 0.001).

TMEM45A mRNA expression was shown to be increased by 5.5 fold in HepG2 cells incubated under hypoxia in the presence or in the absence of etoposide (Figure 4A). This increase correlated with the protective effect of hypoxia against etoposide-induced apoptosis. We then determined that a concentration of 50 nM of TMEM45A siRNA was high enough to specifically inhibit TMEM45A mRNA expression while the RISC-Free control siRNA had no effect.

Caspase 3 activity was then assessed in HepG2 cells transfected with TMEM45A or negative control siRNA before being incubated under normoxia or hypoxia in the presence or in the absence of etoposide. Figure 4B shows that transfection with TMEM45A siRNA significantly increased caspase 3 activity in cells incubated with etoposide under hypoxia, reaching level similar to the one observed in cells transfected with the negative control siRNA and incubated in normoxia with etoposide. No modification was observed in control cells and in cells incubated with etoposide under normoxia. This indicated that the protection conferred by hypoxia requires TMEM45A.

### TMEM45A overexpression is associated with a high risk of breast cancer recurrence

In order to validate these findings, we performed survival analysis using the Kaplan-Meier method on data from a study involving 286 patients with primary breast cancer and available clinical parameters [35]. These patients were allocated to one of two subgroups of 143 patients stratified by TMEM45A expression.

**Figure 5:**
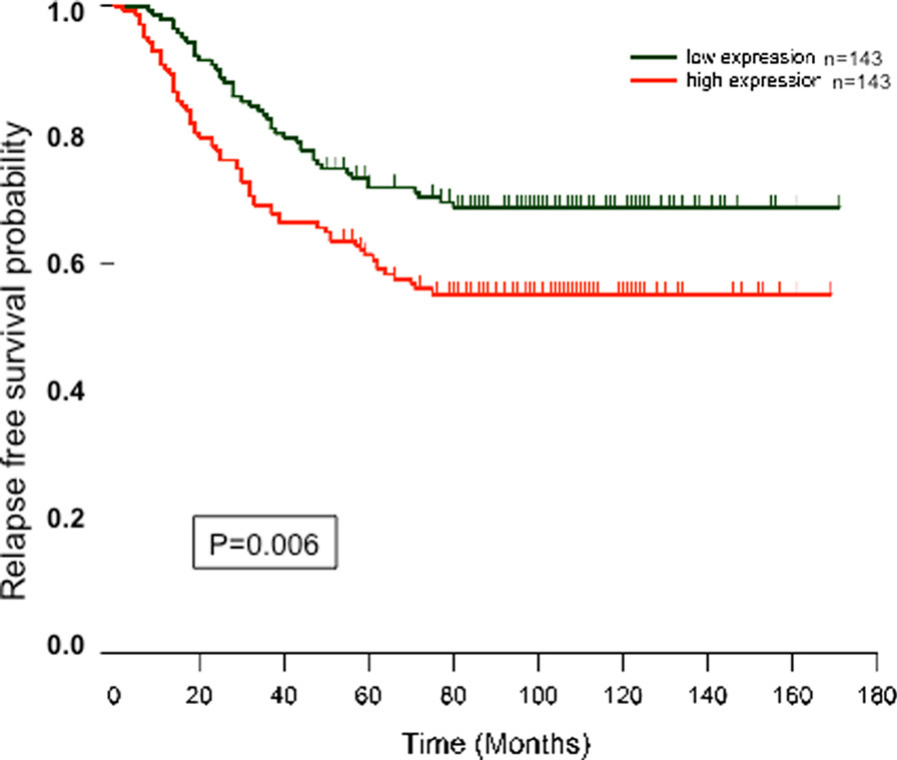
Kaplan-Meier probability of relapse-free survival for 286 patients with lymph-node-negative breast cancer allocated to one of two subgroups stratified by TMEM45A expression.

Kaplan-Meier graph compares the disease-free survival for groups of patients with high and low TMEM45A expression (Figure 5). The results showed that patients whose tumors had high TMEM45A expression had a significantly lower relapse-free survival than those whose tumors had a low TMEM45A expression (p < 0.01). This indicates that TMEM45A may influence cancer cell survival or growth.

### TMEM45A is induced in differentiating keratinocytes

**Figure 6:**
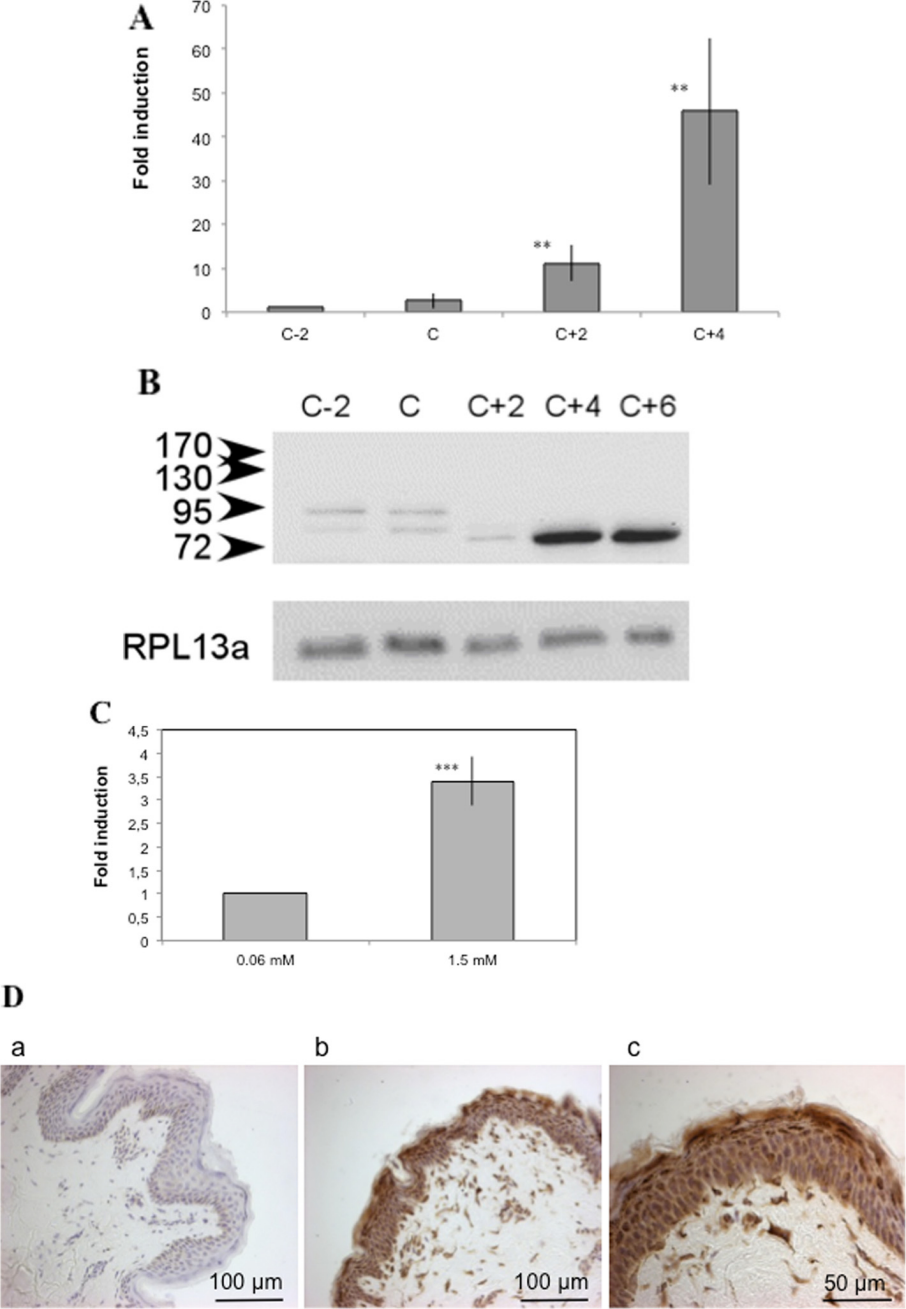
**Analysis of TMEM45A gene expression level in normal human keratinocytes in vitro and in vivo.** (**A** and **B**) Keratinocytes were cultured in autocrine conditions (C-2d, 2 days before culture confluence; **C**, confluence; C + 2d, 2 days after confluence; C + 4d, 4 days after confluence). (**A**) Total RNA has been extracted and retro-transcribed into cDNA. A real time PCR has been performed with specific primers for TMEM45A and for TBP and RPL0 for normalization. Results are expressed as induction level by comparison with the reference condition “C-2d”. Results are expressed as means ± 1 SD for n = 3, keratinocytes obtained from three different normal donors. ** = significantly different from C-2d (p < 0.01). (**B**) TMEM45A protein level was assessed by western blot analysis. RPL13a was used as the loading control. (**C**) Keratinocytes were cultured at low density in the presence of low (0.06 mM) or high (1.5 mM) calcium concentration for 48 hours. Total RNA has been extracted and retro-transcribed into cDNA. A real time PCR has been performed with specific primers for TMEM45A and for TBP and RPL0 for normalization. Results are expressed as induction level by comparison with undifferentiated cells (0.06 mM calcium). Results are expressed as means ± 1 SD for n = 3, keratinocytes obtained from three different normal donors. *** = significantly different from low calcium concentration ((p < 0.001, paired Student’s *t* test). (**D**) Immunohistological staining for TMEM45 in sections of human normal skin section with (b and c) or without primary antibody (a).

MDA-MB-231 and HepG2 cells are from epithelial origin. In both cell lines, TMEM45A plays a role in resistance to apoptosis. On the other hand, differentiated keratinocytes are also known to be resistant to chemotherapeutic agents [36]. We thus sought whether TMEM45A would also be expressed in such normal epithelial cells. Keratinocytes in culture are a good model for that purpose because they can easily be differentiated in culture with a simultaneous growth arrest [37]. TMEM45A expression both at mRNA and protein levels markedly increased between undifferentiated proliferating cells and growth arrested differentiated cells (Figure 6A and B). It has to be noted that the induction of TMEM45A expression in keratinocytes exposed to hypoxia varies according to their differentiation state. TMEM45A expression in keratinocytes at sub-confluence (C-2) is inducible by hypoxia (by 2.92 ± 0.92 fold after 16 hours and by 3.28 ± 0.77 fold after 24 hours, n = 3). However, this is not the case in post-confluent, differentiated keratinocytes: TMEM45A was not induced by hypoxia, probably because its expression was already very high.

Induction of TMEM45A expression was also observed when differentiation was induced by high calcium concentration at low cell density rather than by confluence (Figure 6C) and in vivo as shown by the immunohistological staining performed on human skin sections as indicated by a higher expression of TMEM45A in the upper layers of the epidermis (Figure 6D). TMEM45A expression was not higher in the basal layer despite the fact that the basal layer has been shown to be in a more hypoxic environment [38,39].

## Discussion

Despite new discoveries of the molecular alterations that lead to tumorigenesis and the development of new treatments and therapeutic strategies against cancer, primary treatment failure and relapse after treatment are common and death caused by cancers remains too frequent. The development of resistance to chemotherapy represents significant obstacles to treatment of malignancies relying on conventional cytotoxic agents. Tumor hypoxia is often associated with chemoresistance and with an increased probability of tumor recurrence and death.

MDA-MB-231 breast cancer cells were used for studying the effect of hypoxia on apoptosis induced by paclitaxel and epirubicin, two commonly used chemotherapeutic agents. We observed that hypoxia differentially influenced apoptosis in these cells according to the chemotherapeutic agent. Indeed, in the presence of paclitaxel, apoptosis was inhibited by hypoxic conditions, this was not the case for epirubicin.

Modifications in gene expression induced by an hypoxic environment have already been described to induce resistance to radio- and chemotherapies [40], and although the mechanisms underlying the development of this resistance are partially understood, the most important mechanisms and associated biological pathways remain unknown.

In order to identify mechanisms responsible for the resistance induced by hypoxia, we performed genome-wide gene expression analyses using the Affymetrix technology. We classified the genes whose expression correlated best with drug resistance and i.e. with an induction under hypoxia, with or without paclitaxel, that was lower under normoxia in the presence of paclitaxel. Thirty one genes whose expression meets these criteria were selected. The list of these genes included known hypoxia-responsive targets such as ALDOC [41], PFKFB4 [42], PDK1 [43], BNIP3 [44], NDRG1 [45], ENO2 [46], PFKFB3 [47] and JMJD1A [48]. The list also includes TMEM45A, a gene encoding a putative transmembrane protein of unknown function. TMEM45A was previously found to be upregulated in hypoxia in umbilical cord blood CD133^+^ cells [46]. We showed that TMEM45A expression was also induced by hypoxia in MDA-MB-231 cells, in a HIF-1-dependent manner since HIF-1α silencing prevented the hypoxia-induced upregulation. Sheffer et al. previously showed that TMEM45A was upregulated by zinc, that was used to induce the hypoxic pathway, in colon cancer cells [49]. Benita et al. used combined computational and experimental approaches to show that TMEM45A is a bona fide HIF-1 target gene [34]. In this study, we showed that TMEM45A renders cancer cells resistant to different agents used in chemotherapy. TMEM45A silencing reverted the hypoxia-induced protection against paclitaxel-induced apoptosis in MDA-MB-231 cells. We confirmed the role of TMEM45A in drug resistance, in HepG2 cells exposed to etoposide. In this model, hypoxia also protects cells against etoposide-apoptosis. We found again that the expression profile of TMEM45A paralleled the resistance profile. Finally, its silencing also completely suppressed the protection against etoposide-induced apoptosis. TMEM45A thus exerts an anti-apoptotic function that prevents cell killing.

Similar approaches using small interfering RNA have already been used in order to better understand the regulatory mechanism underlying chemotherapy resistance in various cancer cell types. siRNA directed against Ribophorin II (RPN2), which is part of an N-oligosaccharyl transferase complex, were tested by Honma et al. for their ability to promote apoptosis of docetaxel-resistant MCF7-ADR breast cancer cells and were shown to hypersensitized these cells to the chemotherapeutic agent [50]. Specific siRNA was used to suppress the expression of survivin, a member of the inhibitor of apoptosis protein (IAP) family overexpressed in tumor cells, helping to reverse cisplatin-resistance of A549/DDP multidrug-resistant human lung adenocarcinoma cells [51]. Finally, siRNA-mediated silencing of ERCC1 (excision repair cross complementing group 1) sensitized human colorectal cancer cells to oxaliplatin-induced cell death [52]. Conversely, overexpression of gene associated with resistance decreased sensitivity to drugs. This was for example shown for another transmembrane protein encoding gene, TMEM205. Indeed, an elevated expression of TMEM205 in human cisplatin-resistant cells was associated with cisplatin resistance and ectopic expression of TMEM205 in the cisplatin-sensitive parental cells was shown to confer resistance to cisplatin [53].

The function of TMEM45A is unknown. Our results show that it has an anti-apoptotic function but its mechanism of action remains to be unraveled. Two hypotheses can be proposed: (i) Our results show that TMEM45A protein is induced upon differentiation of keratinocytes, which coincides with an arrest of cell proliferation. Cycling cells are known to be more sensitive to topoisomerase inhibitors such as etoposide, notably because these inhibitors induce less DNA damage in non-dividing cells. However, we show here that TMEM45A is associated with hypoxia-induced resistance and we have previously shown that hypoxia does not prevent etoposide-induced DNA damage, neither does it accelerate their reparation [24]. (ii) TMEM45A is expressed in epithelial-derived cancer cells and highly expressed in differentiated keratinocytes, suggesting that it is involved in the normal function of growth arrested epithelial cells. One family of enzymes typically expressed in epithelia is the transglutaminase family. These enzymes have been associated with chemoresistance by at least two mechanisms: constitutive activation of the focal adhesion kinase and formation of a ternary complex with IκB/p65-p50 leading to activation of the transcription factor NF-κB. Both proteins are known to exert pro-survival activities [54]. A recent work demonstrated that transglutaminase 2 is inducible by hypoxia and suppresses apoptosis by modulating caspase 3 and NF-κB activity [55]. TMEM45A could inhibit apoptosis by promoting the expression of transglutaminase. Alternately, TMEM45A may have a function similar to that of TMEM205. This other transmembrane protein was shown to be co-localized with RAB8, a small endosomal recycling GTPase [56]. Members of this family of GTPases are important regulators of intracellular membrane sorting. In particular, they are thought to mediate membrane transport specificity. Association of TMEM205 with RAB8 could lead to reduced accumulation of cisplatin resulting in cellular resistance to the compound. Whether TMEM45A plays a similar role in MDA-MB-231 and HepG2 cells is not known.

A Kaplan–Meier survival plot was constructed from data from 286 lymph node–negative breast cancer patients who had not received adjuvant systemic treatment. We observed that high TMEM45A expression in primary breast tumors present a higher risk of cancer recurrence.

## Conclusions

Much has been learned in recent years regarding the genetics and molecular pathophysiology of breast cancer. However, chemoresistance is still a heavy burden in cancer patient treatment, and thus it represents a difficult challenge for future improvement of chemotherapy effectiveness. Therefore, a better understanding of the molecular mechanisms of breast cancer resistance should help to improve patient survival. The clinical significance of our findings is that high TMEM45A expression could be used as a molecular prognostic marker to identify patients who have an increased risk of cancer relapse after chemotherapy.

## Methods

### Cell culture and hypoxia incubation

MDA-MB-231 human breast cancer cells and HepG2 human hepatoma cells were maintained in culture in 75-cm2 polystyrene flasks (Costar) with respectively 15 ml of Roswell Park Memorial Institute medium (RPMI 1640, Invitrogen) or Dulbecco’s modified Eagle’s medium (DMEM), containing 10% of fetal calf serum (Invitrogen) and incubated under an atmosphere containing 5% CO_2_.

Primary culture of human skin keratinocytes was performed in autocrine culture as described in Minner et al. [37]. After written informed consent of patients (Dr Bienfait, Clinique St Luc, Namur-Bouge, Belgium), normal human abdominal skin samples were obtained from plastic surgery. The Medical Ethical Committee of Clinique St Luc, Namur-Bouge, approved all described studies, and all experiments were carried out according to the Declaration of Helsinki Principles. In autocrine culture, keratinocytes proliferate until the confluence of the culture is reached concomitantly with cell growth arrest and differentiation. To study high calcium concentration effect, keratinocytes were seeded at very low cell density. The next day, complete medium was changed and replaced by complete medium with 1.5 mM of calcium or normal medium that contains 0.06 mM of calcium.

For hypoxia experiments (1% O_2_), cells were incubated in serum-free CO_2_-independent medium (Invitrogen) supplemented with 1 mM L-glutamine (Sigma) with or without paclitaxel (Invitrogen) at 50 μM, epirubicin (Calbiochem) at 10 μM or etoposide (Sigma) at 50 μM. Normoxic control cells were incubated in the same conditions but in normal atmosphere (20% O_2_).

### siRNA transfection

Silencing of TMEM45A or HIF-1α expression was achieved using siGENOME SMARTpool human TMEM45A (#M-021085-00 containing a mix of 4 siRNA: CAAUGUACUUCUGGAGCUA, GGGAAAUGCUGGACAUCUU, AAGCGAACCUGCUAUCUUG, UAAACAAGGUCACUGGAAU) or HIF1A (#M-004018-05 containing a mix of 4 siRNA: CGUGUUAUCUGUCGCUUUC, GAUGAAAGAAUUACCGAAU, GAUGGAAGCACUAGACAAA, GGACACAGAUUUAGACUUG from Dharmacon. RISC-free control siRNA purchased from Dharmacon was used to control for non-specific effects. For siRNA experiments, 2x10^6^ cells were seeded in 75-cm^2^ polystyrene flasks (Costar) with 10 ml of medium containing 10% of fetal calf serum. Cells were then transfected 24 hours under standard culture conditions with 50 nM siRNA using the DharmaFECT 1 (Dharmacon) transfection reagent according to the manufacturer's instructions.

The transfection media were removed and replaced by culture media for 8 hours. Cells were then trypsinized and seeded at the appropriate density.

### Caspase 3 activity

The fluorogenic substrate Ac-DEVD-AFC was used to measure caspase 3 activity according to Cosse et al. [25].

### LDH release

The Â« cytotoxicity detection kit Â» from Roche Molecular Biochemicals was used to measure lactate dehydrogenase (LDH) release according to the manufacturer’s protocol.

### DNA fragmentation

The measurement of cytoplasmic histone-associated DNA fragments (mono- and oligonucleosomes) after induction of cell death was performed with the Â« cell death detection ELISA Â» (Roche Molecular Biochemicals). Data were normalized for the protein content determined by a BioRad protein assay.

### Visualization of DAPI-labelled nuclei

35,000 cells were seeded on glass coverslips in 24-well culture plates. 24 hours later, cells were incubated under normoxia or hypoxia with or without paclitaxel or epirubicin for 16 hours. Medium was then removed and cells were fixed for 10 min with PBS containing 4% paraformaldehyde and then washed 3 × 5 min in PBS. Cells were then permeabilized with 0.2% Triton X-100 in PBS. After 5 min, 5 μl of DAPI (10 μg/ml) (Sigma, St Louis, USA) were added in well. After 15 min of incubation in dark at 37°C, cells were washed with PBS. Then, the coverslips were mounted in Mowiol (Sigma, St Louis, USA) and observed with a fluorescence microscope at 352 nm.

### Real time RT-PCR

After the incubation, total RNA was extracted using the Total RNAgent extraction kit (Promega). mRNA contained in 2 μg total RNA was reverse transcribed using SuperScript II Reverse Transcriptase (Invitrogen) and oligodT primers according to the manufacturer’s instructions. Forward and reverse primers for TMEM45A were designed using the Primer Express 1.5 software (Applied Biosystem). Amplification reaction assays contained 1x SYBR Green PCR Mastermix (Applied Biosystem) and primers (Applied Biosystems) at the optimal concentrations. RPL13A was used as the reference gene for normalization in MDA-MB-231 and HepG2 cells while the geometric mean of the ct for TBP and RPLP0 was used for keratinocytes and mRNA expression level was quantified using the threshold cycle method.

### Western blot

After the incubation, cell lysates were recovered, proteins were separated by electrophoresis on NuPAGE gels and TMEM45A was revealed by western blot according to Mathay et al. [57]. Anti-TMEM45A antibody was diluted 1/1000 (Sigma, #HPA024082). RPL13 was used as loading control (anti-RPL13 antibody diluted 1/2000, Cell Signaling #27655).

### Immunohistochemical staining of human skin sections

Labeling of section of normal human skin was performed according to Mathay et al. [57], using the same anti-TMEM45A antibody as for the western blot analysis.

### Affymetrix

At the end of the incubation, total RNA was extracted using the Trizol extraction kit (Invitrogen). RNA concentration and purity were determined spectrophotometrically using the Nanodrop ND-1000 (NanoDrop Products). RNA integrity was assessed using an Agilent 2100 Bioanalyser (Agilent). All the steps, including cRNA target preparation, hybridization to Affymetrix HG-U133 Plus 2.0 arrays, washing and array signal acquisition were performed according to the manufacturer’s protocol (Affymetrix) by DNAVision service provider based in Belgium ( http://www.dnavision.com/). Total RNA spiked with bacterial RNA transcript positive controls was converted to double-strand cDNA in a reverse transcription reaction. Sample was then converted and amplified to antisense cRNA, labeled with biotin, in an in vitro transcription reaction. Fragmented biotinylated cRNA and hybridization controls (Affymetrix) were hybridized on array followed by washing and staining steps according to the manufacturer’s procedures. The hybridized probe array was then scanned using the GeneChip® Scanner 3000. Data have been deposited at GEO under accession number GSE39042.

### Statistics

SigmaStat software (Jandle Scientific, Germany) was used for the statistical analysis. Data are presented as means ± SD and were evaluated by one-way ANOVA, using the Holm-Sidak method.

For Affymetrix data normalization, we followed the procedure published by Pierre et al. [58]: probe-level data were normalized and gene expression summaries were computed for each probe set using GeneChip Robust Multichip Analysis (GC-RMA) [59] in the R statistical environment ( http://R-project.org). To process the datasets and in order to avoid inconsistencies, we did not choose CDF (Chip Definition File) but alternative CDFs from AffyProbeMiner, as recommended by Liu et al. [60]. Microsoft Excel was used to calculate average ratios and their standard deviations (S.D.) between CTL condition and the other conditions and between H tax and N tax conditions. p values were also calculated between these conditions. The data set was then filtered for low expression value and calls exhibiting values under an arbitrary threshold value of 8.84 (twice the value of the basal intensity) were considered ‘absent’ otherwise calls were considered ‘present’. Genes were finally considered ‘present’ if they had been assigned a present call in at least two of the three biological replicate samples and could thus be considered to be expressed genes. Fold changes calculated with ‘absent’ gene were considered as ‘qualitative’ difference.

To obtain the dendrogram, unsupervised hierarchical clustering by sample was performed on all probe sets using BRB-ArrayTools developed by Dr. Richard Simon and BRB-ArrayTools Development Team with centered correlation as distance metric and average linkage as linkage function.

For survival analysis, we searched GEO ( http://www.ncbi.nlm.nih.gov/geo/) for studies publishing data with available clinical information. The gene expression data were downloaded from 286 individuals with lymph node–negative breast cancer who had not received adjuvant systemic treatment [35] from GEO (accession number 2034). The downloaded data were GC-RMA normalized in the R statistical environment ( http://R-project.org). Survival analysis was performed using the Kaplan-Meier method and SigmaStat software was used to determine Kaplan-Meier probability of relapse-free survival for the 286 patients allocated to one of two subgroups of 143 patients stratified by TMEM45A expression. The Gehan-Breslow test was used to compare the curves and a p value <0.05 was considered as statistically significant.

## Abbreviations

HIF-1: Hypoxia-inducible factor-1; LDH: Lactate dehydrogenase; TMEM: Transmembrane protein.

## Competing interests

The author(s) declare that they have no competing interests.

## Authors’ contributions

LF, ER and CS carried out the all the experiments on cancer cells and drafted the manuscript. MP participated in the analysis of the microarray data. AH carried out the experiments on keratinocytes. TA, ODB and YP participated in the design of the study. CM conceived of the study, participated in its design and coordination and helped to draft the manuscript. All authors read and approved the final manuscript.

## Pre-publication history

The pre-publication history for this paper can be accessed here:

http://www.biomedcentral.com/1471-2407/12/391/prepub

## Supplementary Material

Additional file 1 Figure S1.Hierarchical clustering of samples data using all genes with centered correlation and average linkage. Samples are clustered on the horizontal axis, with the vertical axis representing the degree of correlation between samples. Click here for file
