# Supplementary figures and images for: TMEM45A is essential for hypoxia-induced chemoresistance in breast and liver cancer cells
Source: BMC Cancer. 2012 Sep 6;12:391. doi: 10.1186/1471-2407-12-391 (PMC3519606; doi:10.1186/1471-2407-12-391)

## Slide 1
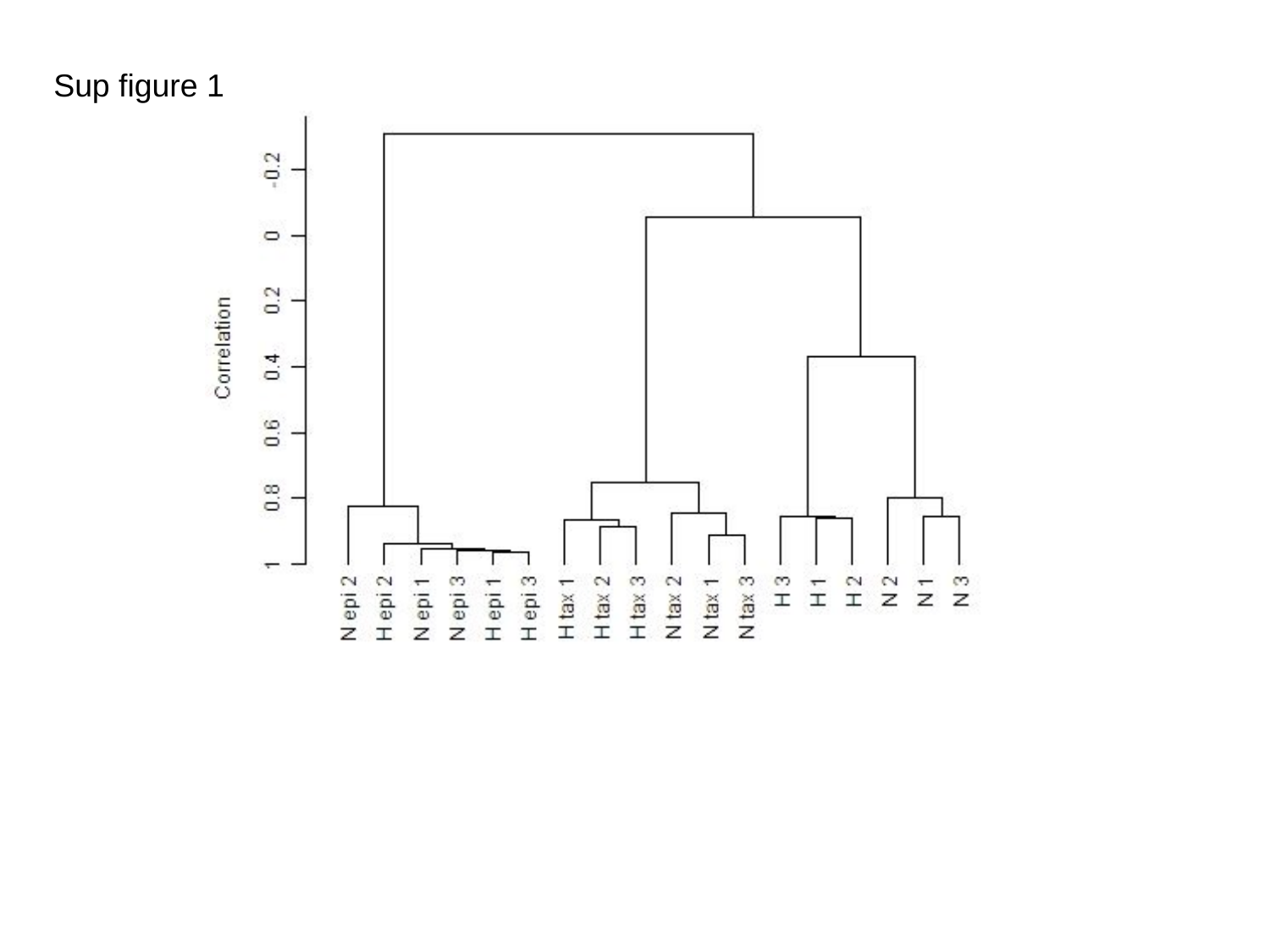

Sup figure 1

Supplement: Additional file 1 — Figure S1.Hierarchical clustering of samples data using all genes with centered correlation and average linkage. Samples are clustered on the horizontal axis, with the vertical axis representing the degree of correlation between samples. [file 1471-2407-12-391-S1.pptx]
